# Chronic GIPR agonism results in pancreatic islet GIPR functional desensitisation

**DOI:** 10.1016/j.molmet.2025.102094

**Published:** 2025-01-07

**Authors:** Iona Davies, Alice E. Adriaenssens, William R. Scott, David Carling, Kevin G. Murphy, James S. Minnion, Stephen R. Bloom, Ben Jones, Tricia M-M. Tan

**Affiliations:** 1 Section of Endocrinology and Investigative Medicine, Imperial College London, United Kingdom; 2 Department of Neuroscience, Physiology, and Pharmacology, University College London, London, United Kingdom; 3 MRC Laboratory of Medical Sciences, London, United Kingdom; 4 Institute of Clinical Sciences, Faculty of Medicine, Imperial College London, London, W12 0NN, United Kingdom

**Keywords:** Glucose-dependent insulinotropic polypeptide receptor (GIPR), Receptor desensitisation, β-arrestin, Receptor internalisation, Obesity, Type 2 diabetes

## Abstract

**Objectives:**

There is renewed interest in targeting the glucose-dependent insulinotropic polypeptide receptor (GIPR) for treatment of obesity and type 2 diabetes. G-protein coupled receptor desensitisation is suggested to reduce the long-term efficacy of glucagon-like-peptide 1 receptor (GLP-1R) agonists and may similarly affect the efficacy of GIPR agonists. We explored the extent of pancreatic GIPR functional desensitisation with sustained agonist exposure.

**Methods:**

A long-acting GIPR agonist, GIP108, was used to probe the effect of sustained agonist exposure on cAMP responses in dispersed pancreatic islets using live cell imaging, with rechallenge cAMP responses after prior agonist treatment used to quantify functional desensitisation. Receptor internalisation and β-arrestin-2 activation were investigated *in vitro* using imaging-based assays. Pancreatic mouse GIPR desensitisation was assessed *in vivo* via intraperitoneal glucose tolerance testing.

**Results:**

GIP108 treatment led to weight loss and improved glucose homeostasis in mice. Prolonged exposure to GIPR agonists produced homologous functional GIPR desensitisation in isolated islets. GIP108 pre-treatment *in vivo* also reduced the subsequent anti-hyperglycaemic response to GIP re-challenge. GIPR showed minimal agonist-induced internalisation or β-arrestin-2 activation.

**Conclusions:**

Although GIP108 chronic treatment improved glucose tolerance, it also resulted in partial desensitisation of the pancreatic islet GIPR. This suggests that ligands with reduced desensitisation tendency might lead to improved *in vivo* efficacy. Understanding whether pancreatic GIPR desensitisation affects the long-term benefits of GIPR agonists in humans is vital to design effective metabolic pharmacotherapies.

## Highlights

• GIP108 is a long-acting, potent and specific GIPR agonist.
• Chronic GIPR agonism results in pancreatic islet GIPR functional desensitisation.
• Internalisation or β-arrestin-2 activation may not underlie mouse GIPR desensitisation.

## 1 Introduction

Obesity is a major global health challenge. There has been great interest in targeting the glucose-dependent insulinotropic polypeptide receptor (GIPR) as a means of augmenting the insulinotropic and anti-obesity action of glucagon-like peptide-1 receptor (GLP-1R) agonists. Whilst GIP's eponymous role in regulating insulin release is clear, the effect on energy balance of GIP remains unclear: both GIPR knockout and GIP-overexpressing mouse models are protected from high-fat diet induced obesity [[1], [2], [3]]. Similarly, pharmacologically targeting the GIPR has yielded conflicting results as, paradoxically, both GIPR agonists and GIPR antagonists produce weight loss in preclinical models of obesity [2,[4], [5], [6], [7]]. Moreover, chemogenetic activation of *Gipr*-expressing neurons in the hypothalamus and hindbrain results in acute anorexia [8,9]. Recent clinical data showed that GIPR agonism produces modest weight loss [10]; on the other hand, there is no reported evidence GIPR antagonism alone produces weight loss in humans. However, consistent with the above-mentioned paradoxical effects, both tirzepatide, a GIPR/GLP-1R dual agonist, and AMG133 (maridebart-cafraglutide), a GIPR antagonist/GLP-1R agonist, produce impressive weight loss and improvements in glycaemia in humans above and beyond GLP-1R agonists alone [11,12].

To explain this paradox, it was proposed by Killion et al. that GIPR agonists and antagonists both produce weight loss because chronic GIPR agonism results in target desensitisation, meaning that GIPR agonists functionally act as antagonists [7]. Since GIPR agonists improve glucose tolerance but antagonists do not, Killion et al. hypothesised that this process differs between tissues, and specifically that greater agonist-induced GIPR desensitisation would be observed in GIPR-expressing anorectic neurons than in pancreatic islets. However, the studies supporting this hypothesis were primarily performed using adipocyte or adipose tissue models and did not utilise tissues relevant to appetite reduction or glycaemic control. Moreover, a recent study described differences in the β-arrestin recruitment and internalisation pattern of GIPRs from different species, showing that the mouse GIPR is less subject to these processes than the human GIPR [13]. The impact of differences in β-arrestin recruitment or internalisation propensity on the extent of functional desensitisation of signalling responses was not examined but is highly relevant to the understanding of both the mechanism of GIPR agonist *versus* antagonist effects and the suitability of the GIPR as a target for “biased” ligands designed to reduce desensitisation [14,15].

In this study, we developed a new long-acting GIPR agonist, GIP108, to facilitate studies of GIPR desensitisation requiring prolonged exposure *in vivo* and *in vitro*. Using this tool, we examined GIPR functional desensitisation *ex vivo* using murine dispersed pancreatic islets and hypothalamic neurons, before investigating the effects of *in vivo* GIPR desensitisation in the context of glycaemic control and appetite reduction.

## 2 Methods

Experimental methods are summarised below, with a full description of certain methodologies found in the supplementary section.

### 2.1 Peptides

GLP-1(7–36)·NH_2_, glucagon(1–29) and human GIP(1–42) were purchased from Bachem, Switzerland; liraglutide from Novo Nordisk; GIP108 from WuXi AppTec, China. GIP108 was designed based on the human sequence of GIP, with amino acid modifications at position 2 (alanine to *2-Aminoisobutyric acid*) and position 14 (methionine to leucine), plus a 20-carbon dicarboxylic acid sited at position 32 via a Lys-γGlu linker. The peptide was synthesized using solid phase peptide synthesis and purified with reverse phase high performance liquid chromatography.

### 2.2 Cell culture

AD-293 cells (Agilent, US) were maintained in Dulbecco's modified medium (DMEM, Thermo Fisher, UK) with 10% foetal bovine serum (FBS, Thermo Fisher, UK) and 1% penicillin/streptomycin (P/S, Sigma, UK) (complete DMEM).

### 2.3 Plasmids

Plasmids encoding full length, wild-type human or mouse GLP-1R, GIPR or glucagon receptor (GCGR) in the pcDNA5/FRT vector (Thermo Fisher, UK), and human or mouse GLP-1R or GIPR featuring an extracellular SNAP-tag distal to the signal peptide in the pcDNA5/FRT/TO vector, were custom synthesised by Genewiz, UK.

### 2.4 HTRF cAMP assays

AD-293 cells were transfected with desired plasmid DNA 24 h prior to cAMP quantification using the cAMP-Gs Dynamic 2 kit (Cisbio, France), as per manufacturer's protocol. Full details are provided in the supplementary methods.

### 2.5 Internalisation and receptor surface expression assays

AD-293 cells were transfected with desired plasmid DNA encoding a SNAP-tagged G-protein coupled receptor (GPCR) 24 h prior to assay. For 1 h internalisation assays, cells were labelled for 10 min with cleavable SNAP-tag probe BG-S-S-649 (New England BioLabs, US). Following 1 h treatment with specified peptide, cells were imaged at baseline and after addition of 2-mercaptoethane sulfonate (Mesna). For 4-hour GPCR surface expression assays, cells were treated with the specified peptide for 4 h, prior to 10-minute labelling with BG-S-S-649 and imaging. Full details are provided in the supplementary methods.

### 2.6 β-arrestin-2 activation assays

AD-293 cells were transfected with desired plasmid DNA encoding a SNAP-tagged GPCR alongside transduction of Bacmam viral particles encoding the Borealis β-arrestin-2 sensor (Montana Molecular, US) and GRK5 (Montana Molecular, US) 24 h prior to assay. Cells were labelled for 10 min with BG-S-S-649 and imaged at baseline and for 30 min following peptide stimulation. Borealis arrestin sensor fluorescence in BG-S-S-649 labelled cells (representing GPCR expressing cells) was quantified and expressed as a % change from baseline. Full details are provided in the supplementary methods.

### 2.7 Animals

All animal procedures were approved by the British Home Office under the UK Animals (Scientific Procedures) Act 1986. Wild type C57BL/6J mice, and mice expressing the cAMP FRET sensor ^T^Epac^VV^ [16,17] in pancreatic beta cells (CAMPER^*Pdx−1-CreERT2*^) and delta cells (CAMPER^*Som-Cre*^) were used. The latter two cohorts were generated by crossing CAMPER mice (C57BL/6-Gt*(ROSA)26Sor*^*tm1(CAG-ECFP*^*^∗^*^*/Rapgef3/Venus*^*^∗^*^*)Kama*^/J; Jax strain #032205) with Pdx1-CreERT2 mice (STOCK Tg(Pdx1-cre/Esr1^∗^)#Dam/J, Jax strain #024968) (to generate CAMPER^*Pdx−1-CreERT2*^) and Sst-Cre mice (STOCK *Sst*^*tm2.1(cre)Zjh*^/J) (to generate CAMPER^*Som-Cre*^). To induce ^T^Epac^VV^ expression in beta cells, harvested islets were treated with tamoxifen for 24 h prior to islet dispersal. Full details of animal husbandry are provided in the supplementary methods.

### 2.8 Pharmacokinetic study

Pharmacokinetic testing of GIP108, administered subcutaneously at 0.5 mg/pig in male large white pigs, was outsourced to Pharmaron Ltd., UK. Full details are stated elsewhere [14].

### 2.9 *In vivo* studies

In all *in vivo* studies, mice were randomised by body weight into groups. Mice received a 50 μL subcutaneous injection of saline (0.9% sodium chloride) or peptide at a specified dose, diluted in saline. Peptide dosing was in nmol per kg of total body weight. For the assessment of the chronic effects of GIP108, mice were injected daily for 15 days. Body composition was measured by EchoMRI on day −1 and day 15. An intraperitoneal glucose tolerance test (IPGTT) (2 g/kg) was conducted on day 14, following 6 h of fasting and 2 h after peptide injection. Non-fasting plasma leptin (ab199082, Abcam, UK), adiponectin (ab226900, Abcam, UK) and amino acids (ab65347, Abcam, UK) were measured on day 16. For the assessments of *in vivo* GIPR desensitisation, mice were injected at 17:00 with vehicle or GIP108 (30 nmol/kg) prior to an acute food intake study/IPGTT/insulin bleeds conducted the following day. Full details of the *in vivo* studies are provided in the supplementary methods.

### 2.10 *Ex vivo* cAMP assays

Primary cultures of mouse hypothalamic neurons [8] and dispersed pancreatic islets [14] were transduced with the Green Up cADDis biosensor (Montana Molecular, US) [18]. Pancreatic islets from CAMPER^*Pdx−1-CreERT2*^ and CAMPER^*Som-Cre*^ mice were not transduced. Cells were pre-treated with peptides as specified, dissolved in culture media. Following 4 h or overnight incubation and a 1-hour washout period, cells were imaged using an epifluorescence microscope at baseline (t = 3 min), following peptide addition (t = 10 min, unless specified otherwise), and following 3-isobutyl-1-methylxanthine (IBMX) (500 μM) and forskolin (FSK) (50 μM) addition (t = 3 min) to maximally stimulate the sensor. In pre-treatment experiments, islet cell fluorescence was normalised to the IBMX/FSK response which was set as 100%. In non-pre-treatment experiments, islet and neuronal cell fluorescence was normalised to both the baseline and IBMX/FSK response. The area-under-the-curve (AUC) for each cell was quantified. Full details of isolation, transduction, plating, imaging and image analysis is provided in the supplementary methods.

### 2.11 Statistics

Analyses was conducted using Prism 10.0 (GraphPad software). Statistical significance was calculated using one sample t test, one- or two-way ANOVA, as indicated in the figure legends. Šídák, Dunnett's and Tukey's post-hoc test were used to correct for multiple comparisons, as indicated in figure legends. Unless specified otherwise, all summarised data points are presented as mean ± SEM. For dose response experiments, 3 parameter fits are plotted. We indicate statistical significance on figures using ∗ = P < 0.05, ∗∗ = P < 0.01, ∗∗∗ = P < 0.001 and ∗∗∗∗ = P < 0.0001.

## 3 Results

### 3.1 GIP108 is a potent and specific GIPR agonist that reduces body weight and improves glycaemia in HFD-induced obese mice

**Figure 1:**
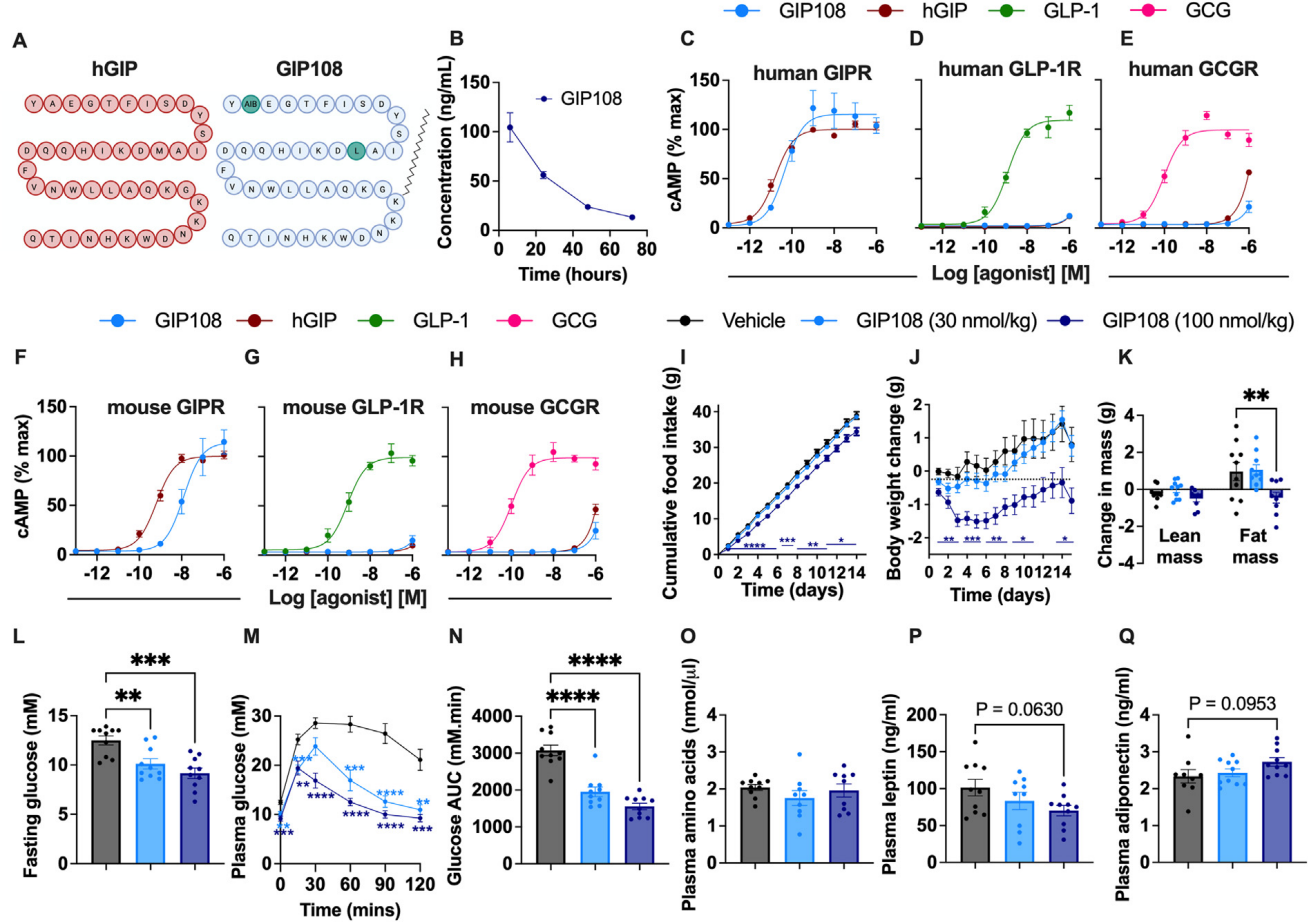
**GIP108 is a GIPR agonist that improves metabolic health in HFD-induced obese mice** **A)** Amino acid sequence of hGIP and GIP108, with green circles in GIP108's sequence representing amino acid differences from hGIP. AIB = 2-aminoisobutyric acid. **B)** Plasma GIP108 concentration (ng/mL) following 0.5 mg injection in pigs (n = 3). **C–H)** cAMP measurements in AD-293 cells transiently transfected with human (**C-E**) and mouse (**F–H**) GIPR (**C** and **F**), GLP-1R (**D** and **G**) and GCGR (**E** and **H**), stimulated for 30 min with indicated peptides, with three-parameter fits shown (n = 3–5). **I-Q**) HFD-induced obese mice received daily afternoon subcutaneous injections of GIP108 at doses of either 30 nmol/kg or 100 nmol/kg, or saline (n = 9–10/group**). I)** Cumulative food intake (g) over 15 days, **J)** body weight change (g) over 15 days, **K)** change in fat and lean mass (g) after 2 weeks, **L)** fasting plasma glucose (mM) measured 2 h after GIP108 or saline injection on day 14, **M)** plasma glucose (mM) and **N)** glucose area-under-the-curve (AUC) measured during intraperitoneal glucose tolerance test 2 h after GIP108 or saline injection on day 14, **O)** plasma amino acids (nmol/μl), **P)** plasma leptin (ng/ml) and **Q)** plasma adiponectin (ng/ml) in the fed state 16 h after their last GIP108 or saline injection. All changes in food intake, body weight and blood glucose at specific time points have been analysed using a two-way ANOVA with time and subgroup as co-variables. Changes in fasting glucose, total area under the curve, body composition and plasma amino acids/hormone levels have been analysed using a one-way ANOVA. Dunnett's test was used to correct for multiple comparisons. All values are displayed as mean ± SEM. ∗ = P < 0.05, ∗∗ = P < 0.01, ∗∗∗ = P < 0.001, ∗∗∗∗ = P < 0.0001. (For interpretation of the references to color in this figure legend, the reader is referred to the Web version of this article.)

As a tool to explore GIPR signalling, we synthesized “GIP108” (Figure 1A), a stabilised GIP analogue based on the human GIP (hGIP) sequence, lipidated to increase pharmacokinetic protraction (Figure 1B). We tested GIP108's *in vitro* GIPR potency and specificity by performing cAMP assays using AD-293 cells transiently transfected with mouse or human GIPR, GLP-1R and GCGR (Figure 1C–H). GIP108 activated human GIPR with potency similar to human GIP(1–42). Potency of both ligands was lower at the mouse GIPR; GIP108 showed a 19.6-fold further lower potency than hGIP, most likely resulting from lipidation and minor changes to the amino acid sequence. GIP108 showed minimal activation of human/mouse GLP-1R and GCGR. Adequate potency, extended half-life and high GIPR specificity indicates it is an appropriate ligand to study GIPR pharmacology.

Next, we assessed whether GIP108 would improve metabolic health in HFD-induced obese mice. Daily subcutaneous injection with GIP108 at 100 nmol/kg (but not 30 nmol/kg) over two weeks significantly lowered food intake (Figure 1I), body weight (Figure 1J) and fat mass, without reductions in lean mass (Figure 1K). While treatment with GIP108 at 30 nmol/kg had no effect on body weight, GIP108 (30 and 100 nmol/kg) resulted in a dose-dependent improvement in fasting glucose and intraperitoneal glucose tolerance (Figure 1L–N), i.e there were weight loss-independent benefits of GIPR agonism at 30 nmol/kg. GIP108 administration had no impact on plasma amino acid levels (Figure 1O) but reduced plasma leptin (Figure 1P) and increased plasma adiponectin (Figure 1Q), both of which are markers of reduced fat mass and improved metabolic health.

### 3.2 Overnight treatment with GIP108 results in *ex vivo* pancreatic GIPR desensitisation

GIP108's ability to potently and specifically activate the GIPR, reduce body weight and improve glycaemia in obese rodent models confirmed that it would be a suitable tool to explore GIPR signalling and functional desensitisation. To explore the potential for tissue-specific desensitisation suggested by Killion et al. [7], we initially wished to explore GIPR desensitisation in hypothalamic neurons, which are known to be important for GIP's anorectic effect [8], with pancreatic islets as a comparator tissue. However, murine hypothalamic neurons transduced with the fluorescence-based cADDis cAMP sensor [18] failed to show detectable responses to hGIP or GIP108 (100 nM and 1 μM), despite significant responses to GLP-1 (10 and 100 nM) (Fig. S1). Thus, our focus shifted to studying GIPR responses in pancreatic islet cells.

As we planned to assess desensitisation effects of both GIP108 and native hGIP using a pre-treat/washout/rechallenge approach, we first aimed to establish suitable agonist concentrations for the different phases of the experiment (Fig. S2). For pre-treatment, we selected 100 nM–1 μM hGIP and 10 nM–1 μM GIP108, as these concentrations produced a detectable signalling response, indicating target engagement, and were pharmacologically active when incubated with cells over 4 h and 24 h, respectively, indicating stability. For rechallenge, 100 nM hGIP and 1 μM GIP108 were selected, which produced peri-maximal responses. For further details on concentration selection, see Supplementary Note.

**Figure 2:**
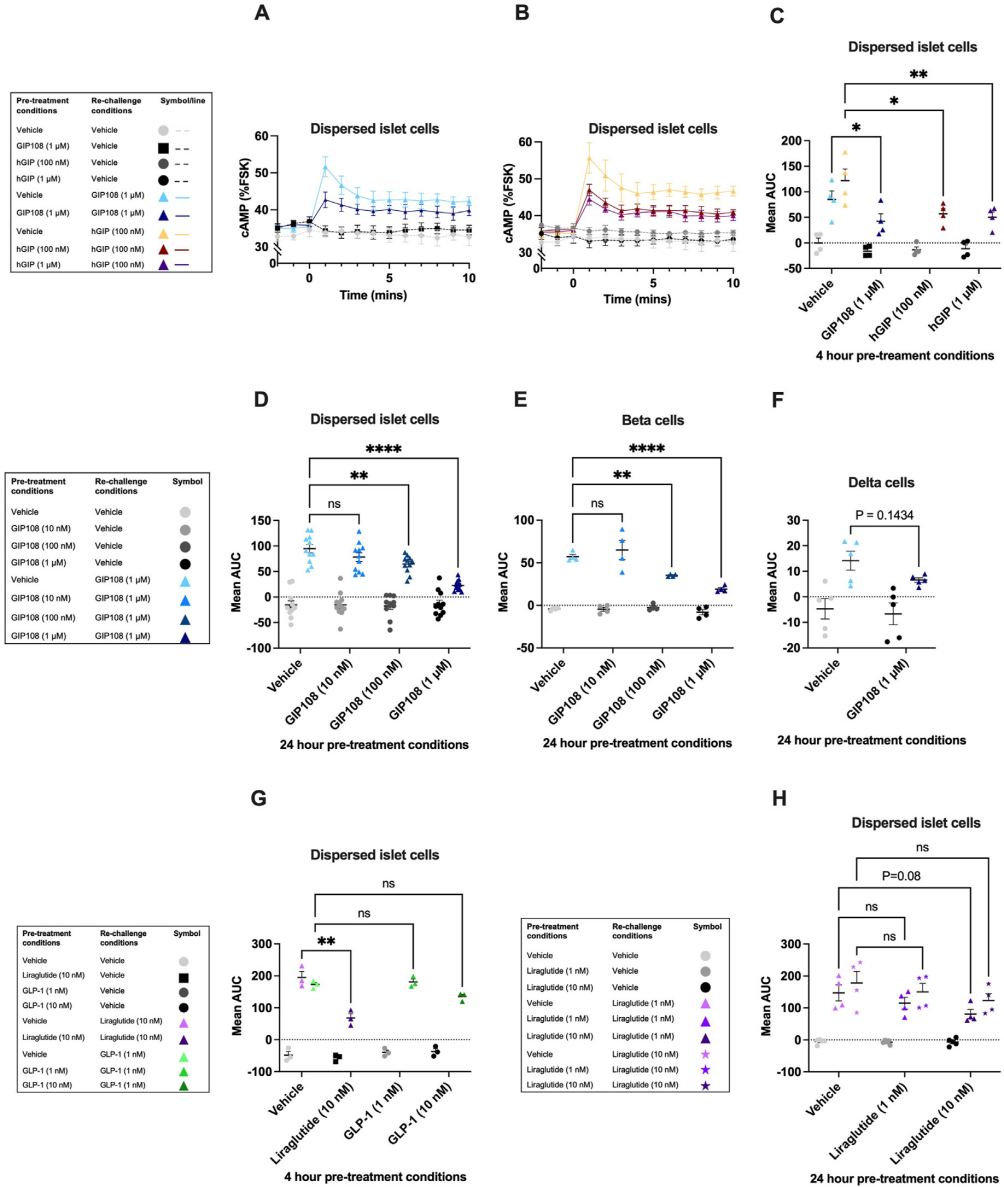
**Prolonged exposure to GIPR agonists results in *ex vivo* GIPR desensitisation in pancreatic islets from lean mice** **A-C)** GIPR desensitisation in all dispersed pancreatic islet cells (transduced with cADDis) (n = 4), following 4 h pre-treatment with specified doses of GIP108 **(A)** and hGIP **(B)**. **D-F)** GIPR desensitisation in all dispersed islet cells (transduced with cADDis) (n = 11–12) (**D**), pancreatic beta cells (CAMPER^*Pdx−1-CreERT2*^) (n = 4) (**E**) and pancreatic delta cells (CAMPER^*Som-Cre*^) (n = 5) (**F**) following 24 h pre-treatment with specified doses of GIP108**. G)** GLP-1R desensitisation in all dispersed pancreatic islet cells (transduced with cADDis) (n = 3) following 4 h pre-treatment with specified doses of liraglutide and GLP-1. **H)** GLP-1R desensitisation in all dispersed pancreatic islet cells (transduced with cADDis) (n = 4–5) following 24 h pre-treatment with specified doses of liraglutide. **A, B)** cAMP profile (as a percentage of the maximum cAMP reached with forskolin (FSK) and 3-isobutyl-1-methylxanthine (IBMX) treatment) following 10 min of treatment with either vehicle or specified peptide, with or without pre-treatment with specified peptide. The cAMP profiles for experiments displayed in **D-H** are displayed in Supplementary Fig. 4. **C–H)** The mean AUC calculated from the cAMP profile for each repeat. Here, the data has been analysed using a two-way ANOVA with pre-treatment groups and re-challenge groups as co-variables. The Šídák test was used to correct for multiple comparisons. All values are displayed as mean ± SEM. ∗ = P < 0.05, ∗∗ = P < 0.01, ∗∗∗ = P < 0.001, ∗∗∗∗ = P < 0.0001.

We examined the effect of prior treatment with hGIP and GIP108 on rechallenge response in dispersed pancreatic islets, with an intervening 60-minute washout period. Interestingly, the rechallenge cAMP response to GIP108 (1 μM) was reduced by approximately 50% when cells had been pre-treated for 4 h with 1 μM of GIP108 (Figure 2A,C). Similarly, 4-hour pre-treatment with hGIP (100 nM and 1 μM) resulted in a 53% and 59% reduction in cAMP production, respectively, following hGIP (100 nM) rechallenge (Figure 2B,C). Next, we investigated whether GIPR desensitisation would be observed following a longer pre-treatment time. 24-hour pre-treatment of islets with hGIP (100 nM and 1 μM) did not reduce the cAMP response to rechallenge with hGIP (100 nM) (Figs. S3A and S4A), likely due to significant peptide degradation (Fig. S2H). However, the cAMP response to GIP108, which was stable during the 24-hour incubation, was significantly reduced when cells had been pre-treated for 24 h with 100 nM or 1 μM of GIP108 (Figures 2D and S4B). The same effects were observed using islets from HFD-induced obese mice (Figs. S3B and S4C). 24-hour GIP108 pre-treatment experiments were repeated using the ^T^Epac^VV^ FRET sensor [16,17] expressed specifically in pancreatic beta cells (using CAMPER^*Pdx−1-CreERT2*^ mice) (Figures 2E and S4D) and delta cells (using CAMPER^*Som-Cre*^ mice) (Figures 2F and S4E), islet cell types with reported *Gipr* expression [19]. GIPR desensitisation was observed in both cell types.

To compare with the GLP-1R, a receptor considered to undergo extensive desensitisation [20,21], we performed analogous experiments using GLP-1 and liraglutide. For pre-treatment, 1 nM–10 nM GLP-1 and 1 nM–10 nM liraglutide were used, taking potency and stability into consideration (Fig. S2). For rechallenge, 1 nM of GLP-1 and 1 nM–10 nM liraglutide were used. For further details on dose finding, see Supplementary Note. Following 4 h of pre-treatment with liraglutide (10 nM) and GLP-1 (1 nM and 10 nM), significant GLP-1R desensitisation was observed with liraglutide pre-treatment (Figures 2G and S4F). While GLP-1 (10 nM) induced partial GLP-1R desensitisation, this was not statistically significant (Figures 2G and S4G). Following 24 h of pre-treatment with liraglutide (1 nM and 10 nM), GLP-1R desensitisation was observed in dispersed islet cells at 10 nM when re-stimulated with liraglutide (1 nM) (Figures 2H and S4H, I).

We next wished to determine whether the desensitising effect of GIP108 (Figs. S3C and S4J) was specific. This appeared to be the case as cAMP responses to the β_2_-adrenergic receptor agonist isoproterenol (1 μM) remained intact (Figs. S3D and S4K). There was, however, a small but significant decrease in response to GLP-1 (100 nM) following GIP108 (1 μM) pre-treatment (Figs. S3E and S4L), suggestive of partial heterologous desensitisation. To probe this further, we performed analogous experiments pre-treating cells with liraglutide (10 nM). Interestingly, while significant GLP-1R desensitisation was observed when re-stimulated with liraglutide (1 nM) (Figs. S3F and S4M), responses to GIP108 (1 μM) were not compromised by liraglutide pre-treatment (Figs. S3G and S4N).

Overall, these data indicate prolonged activation results in desensitisation of pancreatic GIPR signalling *ex vivo*. Moreover, whilst direct comparisons carry caveats related to relative target engagement and ligand stability, the degree of desensitisation at the mouse GIPR was similar to that at the GLP-1R when using ligand concentrations selected to produce a similar acute signalling response.

### 3.3 Functional desensitisation of the mouse GIPR is independent of β-arrestin-2 activation or receptor internalisation

**Figure 3:**
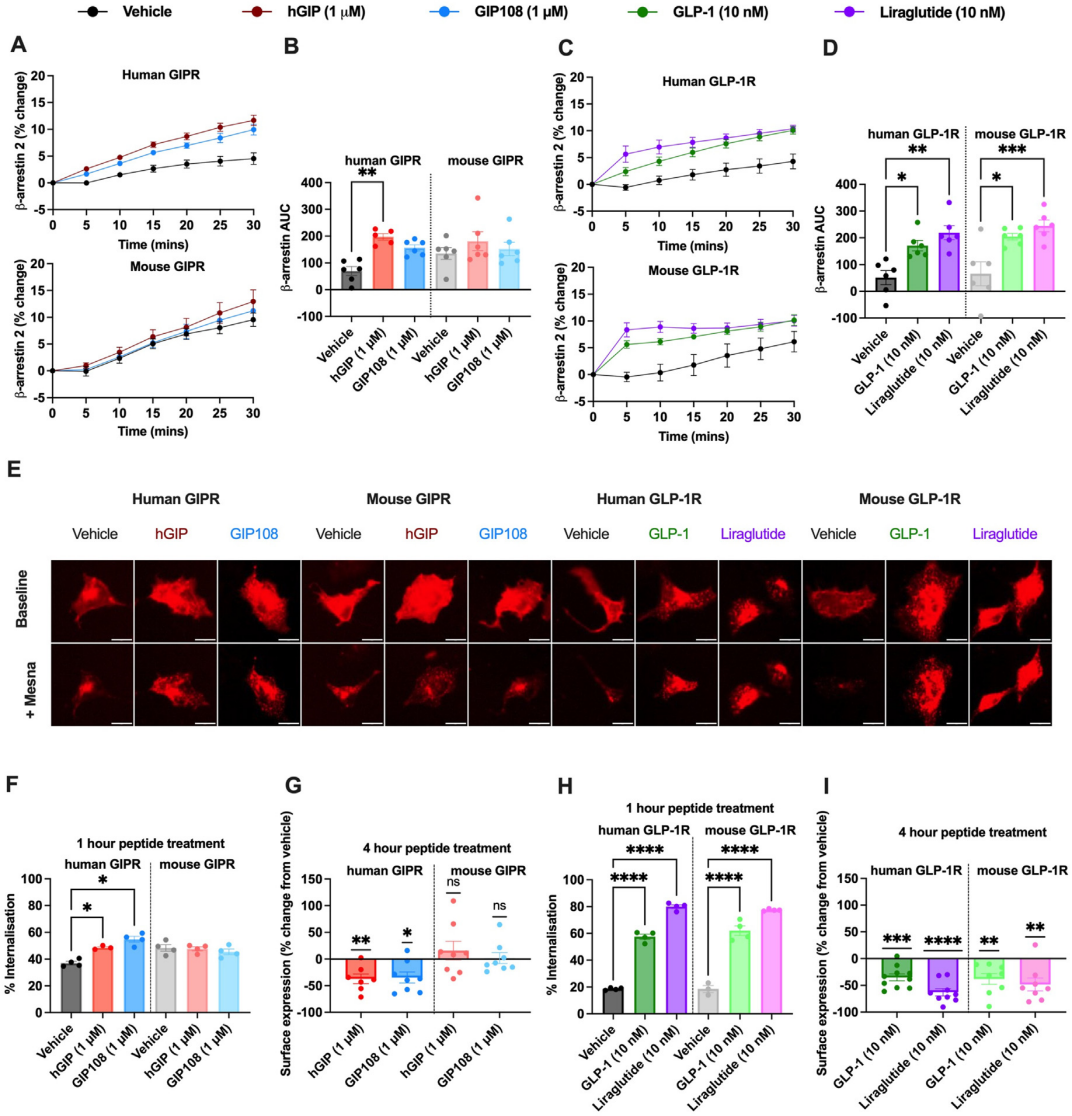
**Prolonged GIPR agonism does not result in increased mouse GIPR β-arrestin-2 activation or internalisation** β-arrestin-2 activation, receptor expression and internalisation assays conducted in AD-293 cells transduced with SNAP-tagged human and mouse GIPR treated with hGIP (1 μM) and GIP108 (1 μM) **(A, B, F, G)** and SNAP-tagged human and mouse GLP-1R treated with GLP-1 (10 nM) and liraglutide (10 nM) **(C, D, H, I). A, C)** % change in β-arrestin-2 activation following 30-minute stimulation with specified peptides (n = 5–6). **B, D)** β-arrestin-2 activation AUC generated from the corresponding time courses in **A** and **C**, significance tested using a one-way ANOVA with Tukey's test used to correct for multiple comparisons. **E)** Representative images (scale bar: 20 μm) of experiments in **F** and **H**. **F, H)** 1-hour peptide treatment measuring % internalisation (n = 3–4), significance tested using a one-way ANOVA with a Šídák test used to correct for multiple comparisons. **G, I)** 4-hour peptide treatment measuring receptor surface expression as a % change from vehicle treated cells (n = 7–8), significance tested using a one-sample T test. All values are displayed as mean ± SEM. ∗ = P < 0.05, ∗∗ = P < 0.01, ∗∗∗ = P < 0.001, ∗∗∗∗ = P < 0.0001.

Given the GIPR and GLP-1R functional desensitisation observed in *ex vivo* dispersed mouse islet cell models, we aimed to dissect the roles of known negative regulators of receptor signalling in these effects. The β-arrestins were considered likely candidates as they sterically hinder G protein activation and promote receptor downregulation through coupling to endocytosis [22,23]. Indeed, the duration of human GIPR-mediated cAMP signalling is enhanced in the absence of β-arrestins [24]. We directly compared β-arrestin-2 conformational activation kinetics in response to hGIP (1 μM) and GIP108 (1 μM) by the human and mouse SNAP-tagged GIPRs expressed in AD-293 cells, and GLP-1 (10 nM) and liraglutide (10 nM) at the corresponding GLP-1Rs (Figure 3A–D). Whilst hGIP (1 μM) resulted in significant increases in β-arrestin-2 activation at the human SNAP-tagged GIPR compared to vehicle control, neither hGIP (1 μM) nor GIP108 (1 μM) resulted in significant increases in β-arrestin-2 activation at the mouse SNAP-tagged GIPR (Figure 3A,B). In contrast, GLP-1 (10 nM) and liraglutide (10 nM) both resulted in significant β-arrestin-2 activation at the human and mouse SNAP-tagged GLP-1R compared to vehicle control (Figure 3C,D).

We also measured patterns of agonist-induced internalisation of the SNAP-tagged human and mouse GIPR and GLP-1R, using a reversible, pre-stimulation labelling approach to directly quantify the amount of receptor internalised over a 60-minute stimulation period (Figure 3E,F, H), and post-stimulation labelling to quantify loss of surface receptor after a 4-hour stimulation (Figure 3G,I). At 1-hour post agonist treatment, both hGIP (1 μM) and GIP108 (1 μM) increased human GIPR internalisation compared to vehicle but did not increase mouse GIPR internalisation (Figure 3F). Similarly, at 4 h post treatment with hGIP (1 μM) and GIP108 (1 μM), there was a reduction in residual human GIPR but not in mouse GIPR detectable at the cell surface (Figure 3G). In comparison, at both 1-hour and 4-hours post peptide treatment, GLP-1 (10 nM) and liraglutide (10 nM) significantly increased both mouse and human GLP-1R internalisation compared to vehicle (Figure 3H,I). Notably, GIPR internalisation during vehicle treatment was greater than for GLP-1R (Figure 3F,H), particularly with mouse GIPR, in keeping with a higher constitutive activity.

Therefore, our results are in agreement with a recent study demonstrating minimal β-arrestin and internalisation responses at the mouse GIPR [13], suggesting the existence of alternative mechanisms driving the substantial functional desensitisation seen with prolonged GIPR agonism in mouse islet models.

### 3.4 Overnight treatment with GIP108 results in pancreatic, but not CNS, GIPR desensitisation *in vivo*

**Figure 4:**
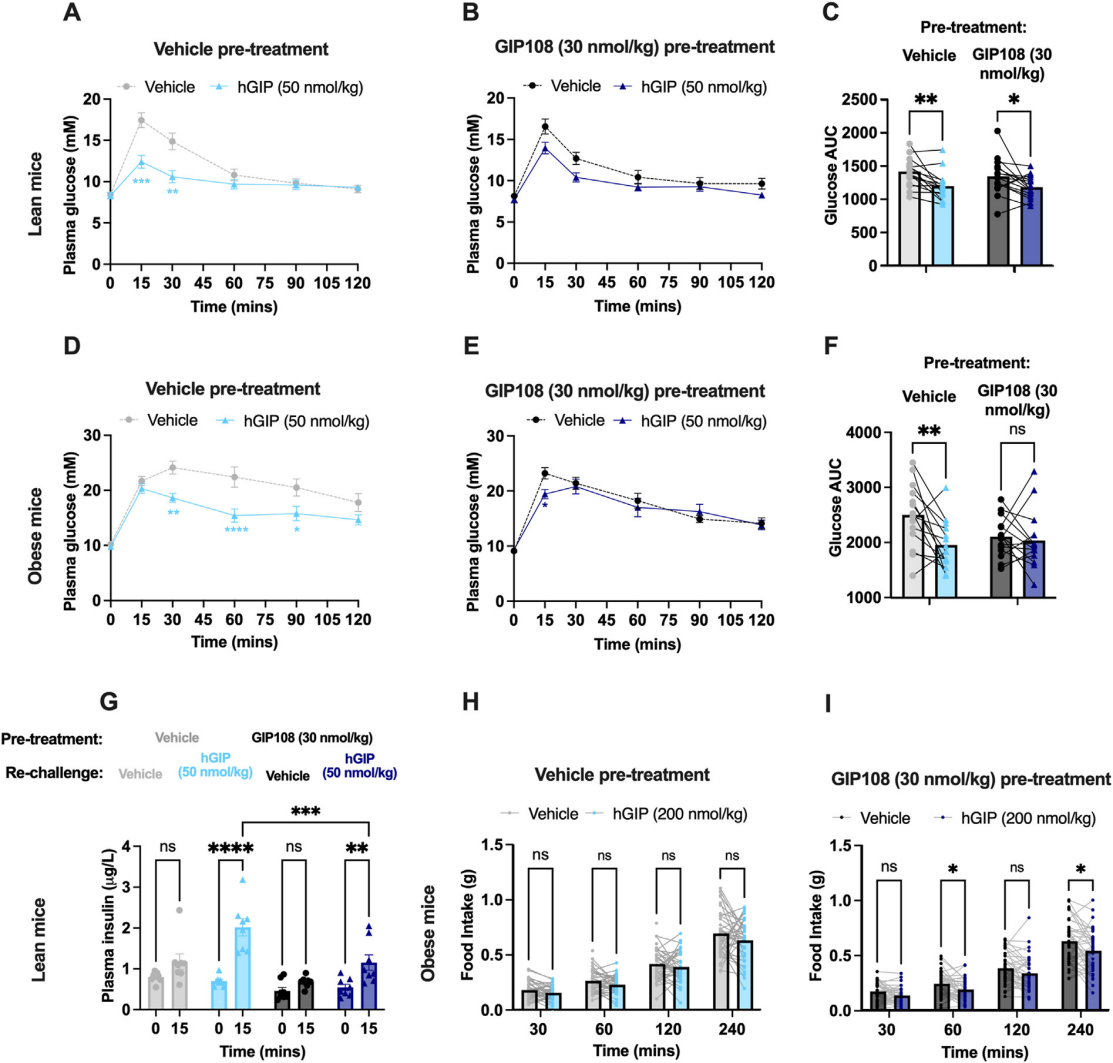
**GIP108 pre-treatment results in pancreatic but not CNS GIPR desensitisation *in vivo***. **A-F)** 4-way crossover 120-minute intraperitoneal glucose tolerance tests testing responses to vehicle and hGIP (50 nmol/kg) in lean (**A-C**) (n = 15–16) and HFD-induced obese mice (**D-F**) (n = 14–15). **G)** 2-way crossover study measuring plasma insulin (ug/L) at baseline and 15 min following glucose injection ± hGIP (50 nmol/kg), following vehicle or GIP108 (30 nmol/kg) injection 21 h prior to blood sampling in lean mice (n = 8). **H,I)** 4-way crossover fast-refeed food intake studies testing responses to vehicle and hGIP (200 nmol/kg) in HFD-induced obese mice (n = 37–40). Mice had received a subcutaneous vehicle injection (**A**, **D, H**) or GIP108 (30 nmol/kg) injection (**B**, **E, I**) 15 h prior to the food intake study and 21 h prior to the IPGTT. **A**, **B**, **D**, **E**) Plasma glucose time course following glucose and peptide injection at t = 0 min. **C**, **F**) Mean glucose AUC generated from the time course. Blood glucose and food intake at specific time points have been analysed using a two-way ANOVA with time and subgroup as co-variables. The Šídák test was used to correct for multiple blood glucose comparisons and Tukey's test was used to correct for multiple food intake comparisons. Glucose AUCs and insulin values have been analysed using a two-way ANOVA with glucose treatment and pre-treatment groups as co-variables. The Šídák test was used to correct for multiple comparisons. Values are displayed as mean (± SEM in time courses and insulin values). ∗ = P < 0.05, ∗∗ = P < 0.01, ∗∗∗ = P < 0.001, ∗∗∗∗ = P < 0.0001.

To test the physiological relevance of our *ex vivo* findings, we explored whether GIP108 pre-treatment would reduce hGIP's ability to improve glucose tolerance during an intraperitoneal glucose tolerance test (IPGTT) in both lean and HFD-induced obese mouse models (Figure 4A–F). In lean and HFD-induced obese mice that received a subcutaneous injection of vehicle 21 h prior to an IPGTT, hGIP (50 nmol/kg) significantly improved glucose tolerance. However, in mice that received pre-treatment with GIP108 (30 nmol/kg), hGIP (50 nmol/kg)'s ability to improve glucose tolerance was reduced. The plasma insulin response to GIP/glucose administration was also blunted after GIP108 (30 nmol/kg) pre-treatment (Figure 4G), suggesting that these glycaemic effects were the result of beta cell GIPR desensitisation. Moreover, a higher dose of hGIP (500 nmol/kg) was still able to improve glucose tolerance in lean and obese mice pre-treated with GIP108 (30 nmol/kg) (Fig. S5), indicating that the blunted response at the lower hGIP dose was not because GIP108 had already produced a maximal improvement in glucose tolerance.

Finally, we tested whether pre-treatment with GIP108 (30 nmol/kg) would attenuate the anorectic effects of hGIP during an acute fast-refeed study in HFD-induced obese mouse models (Figure 4H,I). For rechallenge, hGIP (200 nmol/kg) was chosen as an acutely anorectic dose (Fig. S6). GIP (200 nmol/kg) produced modest reductions in food intake in both vehicle and GIP108 (30 nmol/kg) pre-treated mice, reaching significance at t = 60 and t = 240 in the GIP108 pre-treated mice. Thus, we did not see evidence for CNS GIPR desensitisation following prolonged agonist stimulation.

## 4 Discussion

In summary, we have shown that sustained stimulation of the GIPR results in both *ex vivo* and *in vivo* pancreatic GIPR desensitisation. Additionally, to our knowledge, this is the first study to explore the differences between CNS and pancreatic GIPR desensitisation *in vivo*. These findings are relevant in the context of ongoing debate about why both GIPR agonists and antagonists paradoxically produce weight loss, with GIPR desensitisation in anorectic neurons proposed as a potential mechanism. Moreover, our results contribute to the knowledge base underpinning efforts to improve the targeting of class B GPCRs using biased agonists designed to promote long-lasting signalling by reducing β-arrestin-mediated desensitisation.

GIP108 was used throughout this study as a long-acting specific GIPR agonist. *In vitro* pharmacological testing showed that GIP108 had a lower potency at the mouse GIPR than native hGIP, which we suspect is at least partially due to the effects of the 20-carbon lipid side chain shared with tirzepatide (which also shows disproportionately reduced mouse GIPR signalling) [25]. Nevertheless, significant improvements in body weight and glycaemia observed in HFD-induced obese mice following GIP108 administration showed that *in vivo* efficacy of the ligand was maintained, likely through improved pharmacokinetics of the compound. Thus, we concluded it was an appropriate ligand to study the effects of prolonged GIPR agonism on receptor desensitisation.

Our original aim was to compare *ex vivo* pancreatic and hypothalamic GIPR desensitisation. However, cAMP responses were barely detectable in hypothalamic neurons when treated with GIPR ligands, despite clear responses to GLP-1. This is most likely to be due to a low proportion of GIPR-expressing neurons, which in turn themselves have a relatively low GIPR density [8]. Moreover, GIPR expression could have been reduced by the high glucose content of the neurobasal culture medium, as high glucose levels have been shown to downregulate *Gipr* expression in β-cells [26]. Models to demarcate Gipr-positive cells exist, such as *Gipr*-Cre mice, but as the transgene disrupts the endogenous *Gipr* locus, only heterozygous mice can be used for functional studies, which is likely to affect GIP-mediated desensitisation responses due to a gene dosage effect [8]. Therefore, we decided to focus on pancreatic GIPR desensitisation for *ex vivo* studies. 24-hour pre-treatment of dispersed pan-islet cells, beta cells and delta cells with GIP108 resulted in significant GIPR desensitisation. 4-hour treatment of dispersed islet cells with hGIP also resulted in significant GIPR desensitisation. We acknowledge a limitation in that cAMP was the only functional *ex vivo* readout used in our study, and future work to measure glucagon and insulin secretion would be of merit. Nevertheless, our observations led us to ask the following questions: 1) how does pancreatic islet GIPR desensitisation compare to GLP-1R desensitisation, 2) what is mediating the observed GIPR desensitisation and 3) is GIPR desensitisation observed *in vivo*?

In answer to the first question, we observed significant *ex vivo* islet GLP-1R desensitisation when the cells were pre-treated with liraglutide for 4 and 24 h. We are unable to directly compare the magnitude of receptor desensitisation between the GIPR and GLP-1R as the responses seen could be agonist specific. Moreover, whilst matched to achieve similar acute signalling responses, the GIPR and GLP-1R agonist doses were not matched for receptor occupancy. Nevertheless, our data clearly demonstrate that both mouse GIPR and GLP-1R are subject to functional desensitisation in the context of sustained agonist exposure.

Next, we asked whether the *ex vivo* GIPR desensitisation was a result of β-arrestin-2 activation and/or GIPR internalisation. In line with a recent study highlighting species-specific GIPR pharmacology [13], GIP108 and hGIP barely produced any β-arrestin or internalisation responses at the mouse GIPR in AD-293 cells, although both were detected with the human GIPR (and both GLP-1R species). This is noteworthy for multiple reasons. First, if the human GIPR activates β-arrestin-2 and internalises more than the mouse GIPR in response to agonist stimulation, perhaps the pancreatic GIPR desensitisation observed here in mouse models would be even more apparent in human studies. Second, while β-arrestin-2 recruitment is typically considered to be an “off-switch” to GPCR signalling, two recent studies identified β-arrestin-2 as a vital positive mediator in GIPR signalling [27,28]. Thus, whether G-protein biased GIPR agonists would have enhanced beneficial glycaemic effects remains uncertain. Third, in the potential absence of β-arrestin-2 recruitment or internalisation, other possible drivers of mouse GIPR desensitisation could include β-arrestin-1 recruitment, second messenger dependent kinases such as PKA and PKC, and regulators of G-protein signalling (RGS) proteins [[29], [30], [31]]. The latter two possibilities could explain the heterologous desensitisation observed at the GLP-1R following GIP108 pre-treatment, whereas the lack of heterologous desensitisation observed at the GIPR following liraglutide pre-treatment suggests differences in the desensitisation mechanism of the GLP-1R. Additional studies are required to elucidate the exact molecular mechanisms of GIPR desensitisation in pancreatic islets.

Lastly, we provide evidence that pancreatic GIPR desensitisation is also present *in vivo*, as mice that received GIP108 pre-treatment no longer exhibited improvements in glucose tolerance when administered hGIP during an IPGTT. Interestingly, we did not observe *in vivo* CNS GIPR desensitisation as hGIP still acutely reduced appetite in mice that had received GIP108. We acknowledge that this effect size is small at the dose of hGIP chosen, and the use of mGIP in future acute food intake studies may be preferable [32]. The lack of observed CNS GIPR desensitisation could be explained by differential desensitisation of pancreatic GIPR vs CNS GIPR. Also, while we chose the dose of GIP108 to match the dose used prior in IPGTTs, it is plausible that *in vivo* CNS GIPR desensitisation would have been revealed at a higher pre-treatment dose. However, it is possible that higher doses of GIP108 could have significantly reduced food intake to the point at which no further reductions in appetite via GIPR signalling would have been possible.

Despite not being able to directly compare *ex vivo* GIPR desensitisation between pancreatic and neuronal tissue, this study still provides insight into why GIPR agonists and GIPR antagonists both reduce food intake. The widely considered GIPR desensitisation paradigm states that chronic GIPR agonism results in receptor desensitisation leading GIPR agonists to act as functional antagonists [7]. If true, one would expect acute GIPR agonism to have an orexigenic effect. However, in keeping with other reports [2,8,33], we observe GIP's anorectic effect within 30 min following injection, suggestive that acute GIPR agonism is truly anorectic. Other suggested mechanisms of why GIPR agonists and antagonists reduce food intake, such as potentially acting on different neuronal populations to mediate their effects, discussed extensively elsewhere, still require experimental verification [34].

Finally, it is important to highlight that, despite observing pancreatic GIPR desensitisation *ex vivo* and *in vivo*, chronic treatment with GIP108 still improved glucose tolerance. This could either be because there is less GIPR desensitisation when agonist exposure is longer than 24 h or that GIP108 is a more powerful incretin *in vivo* than hGIP (50 nmol/kg) due to its lipidation and longer half-life. Nevertheless, this of course does not mean a ligand with reduced desensitisation tendency would not lead to improved *in vivo* efficacy. There is compelling evidence that GLP-1R agonists causing reduced desensitisation provide longer lasting glycaemic benefits than their unbiased counterparts [14]. The development of GIPR agonists that induce less desensitisation is a worthy field of future research [15], but it remains unclear whether reducing β-arrestin responses will provide similar benefits at GIPR as seen for GLP-1R – at least when testing in mice. Ultimately, whether pancreatic GIPR desensitisation impacts the long-term benefits of GIPR agonists in humans remains unknown and is an important topic for further study.

## CRediT authorship contribution statement

**Iona Davies:** Writing – review & editing, Writing – original draft, Methodology, Investigation, Formal analysis, Data curation, Conceptualization. **Alice E. Adriaenssens:** Methodology. **William R. Scott:** Supervision. **David Carling:** Writing – review & editing, Supervision, Resources. **Kevin G. Murphy:** Writing – review & editing, Resources. **James S. Minnion:** Resources. **Stephen R. Bloom:** Resources. **Ben Jones:** Writing – review & editing, Writing – original draft, Methodology, Investigation, Formal analysis, Data curation, Conceptualization. **Tricia M-M. Tan:** Writing – review & editing, Writing – original draft, Supervision, Conceptualization.

## Declaration of competing interest

The authors declare the following financial interests/personal relationships which may be considered as potential competing interests: Tricia MM Tan reports a relationship with Zihipp Ltd that includes: consulting or advisory. Stephen R Bloom reports a relationship with Zihipp Ltd that includes: employment. James S Minnion reports a relationship with Zihipp Ltd that includes: employment. Ben Jones reports a relationship with Metsera Inc that includes: consulting or advisory. Ben Jones reports a relationship with Eli Lilly and Company that includes: funding grants. Ben Jones reports a relationship with Sun Pharmaceutical Industries Ltd that includes: funding grants. Stephen R Bloom has patent #PCT/GB2024/050504 licensed to IP2IPO INNOVATIONS LIMITED. If there are other authors, they declare that they have no known competing financial interests or personal relationships that could have appeared to influence the work reported in this paper.

## Data Availability

Data will be made available on request.
