# Supplementary material for: Chronic GIPR agonism results in pancreatic islet GIPR functional desensitisation
Source: Mol Metab. 2025 Jan 7;92:102094. doi: 10.1016/j.molmet.2025.102094 (PMC11786100; doi:10.1016/j.molmet.2025.102094)
Supplement: Multimedia component 1 [file mmc1.docx]

**Supplementary methods**

**HTRF cAMP assays**

AD-293 cells were added to a 12-well plate and maintained in 2 ml complete DMEM. 1 μg of specified plasmid DNA was added to each well alongside Lipofectamine 2000 (Thermo Fisher, UK). cAMP production was quantified 24 hours following transfection using homogenous time resolved fluorescence (HTRF), specifically the cAMP-Gs Dynamic 2 kit (Cisbio, France). Peptides and cells were diluted in DMEM + 1% P/S + 0.1% BSA. For peptide stability testing, cells, fresh peptide and peptide incubated with dispersed islet cells were diluted in RPMI-1640 (Thermo Fisher, UK) + 10% FBS + 1% P/S. 10 μl of peptide and 10 μl of cell suspension were added in duplicate to an opaque 96-well plate (Grenier Bio-One, Austria) and incubated at 37°C for 30 minutes. 10 μl of cAMP-d2 and anti-cAMP cryptate, both diluted in lysis buffer, were added in quick succession as per the manufacturer’s protocol. The plates were then incubated at room temperature for 45 minutes and read using the SpectraMax i3x (Molecular Devices, US).

**Internalisation assays**

AD-293 cells were added to a 96-well plate and maintained in 200 μl complete DMEM. 150 ng of mouse or human SNAP-tagged plasmid GIPR DNA, or 100 ng of mouse or human SNAP-tagged plasmid GLP-1R DNA was added to each well alongside Lipofectamine 2000 (Thermo Fisher, UK) and left for 24 hours.

For 1 hour internalisation assays, cells were labelled with cleavable SNAP-tag probe BG-S-S-649 (New England BioLabs, US) in complete DMEM for 10 minutes prior to washing and 1 hour treatment with specified peptide, reconstituted in DMEM + 0.1% BSA. Cells were washed and 100 μl of TNE buffer (NaCl 100 mM, EDTA 1 mM, Tris HCl 50 mM; pH 8.6) was added. Cells were imaged using an automated epifluorescence microscope (modified Nikon Ti-2E with LED illumination). Cells were imaged at baseline and after addition of 2-mercaptoethane sulfonate (Mesna, 100 mM in TNE buffer) to cleave residual surface GPCR. GPCR internalisation was quantified as the cell fluorescence following Mesna addition (representing internalised GPCR), corrected for the baseline cell fluorescence (representing total cellular GPCR).

For 4-hour GPCR surface expression assays, cells were treated with specified peptide for 4 hours in complete DMEM prior to labelling with BG-S-S-649. Cells were washed and imaged in TNE buffer. GPCR surface expression following peptide treatment was quantified as cell fluorescence as a percentage of cell fluorescence following vehicle treatment.

**β-arrestin-2 activation assay**

AD-293 cells were added to a 96-well plate and maintained in 200 μL complete DMEM. 150 ng of mouse or human SNAP-tagged plasmid GIPR or GLP-1R DNA, 2 μL of Borealis β-arrestin-2 sensor (Montana Molecular, US) and 2 μL of GRK5 (Montana Molecular, US) was added to each well alongside Lipofectamine 2000 and left for 24 hours.

Cells were labelled with cleavable SNAP-tag probe BG-S-S-649 in complete DMEM for 10 minutes prior to washing and imaging. Cells were imaged in Krebs buffer with 6 mM glucose and 0.1% BSA at 37°C using an automated epifluorescence microscope (modified Nikon Ti-2E with LED illumination). Cells were imaged at baseline, registering both BG-S-S-649 fluorescence and borealis arrestin sensor fluorescence. Borealis arrestin sensor fluorescence was then imaged for 30 minutes after peptide addition in intervals of 5 minutes. Borealis arrestin sensor fluorescence in BG-S-S-649 labelled cells was quantified using Fiji v1.54f (NIH) and expressed as a % change from baseline.

**Animals**

All animal procedures were approved by the British Home Office under the UK Animals (Scientific Procedures) Act 1986. For *in vivo* studies, male C57BL/6J mice (10-12 weeks; Charles River, UK) were singly housed in individually ventilated cages with a standard 12:12 hour light-dark cycle. Unless fasted, mice had free access to food pellets (Lean mice: RM1 - Special Diet Services, UK, high fat diet (HFD) induced obese mice: 60% kcal fat diet, D12492, Research Diets, Inc. New Brunswick, US), and water. For studies involving HFD-induced obese mice, mice were maintained on HFD for at least 8 weeks prior to the initiation of studies. For *ex vivo* islet and neuronal studies, male and female 6–25 week old C56BL/6J mice (Charles River, UK), CAMPER*^Pdx~~-~~1-CreERT2^*   and CAMPER*^Som-Cre^* mice were used.

**Peptide dosing**

In all *in vivo* studies, mice were randomised by body weight into groups. Mice received a 50 μL subcutaneous injection of saline (0.9% sodium chloride) or peptide at a specified dose, diluted in saline. Peptide dosing was in nmol per kg of total body weight.

**Chronic GIP108 administration to HFD-induced obese mice**

HFD-induced obese mice (mean starting weight of 43.4 g) received daily injection of saline, GIP108 (30 nmol/kg) and GIP108 (100 nmol/kg) (n=9-10/group) for 15 days. Mice were injected between 16:00 and 17:00, before the beginning of the natural feeding period. Food intake and body weight were measured daily for the entirety of the study. Fat mass and lean mass were calculated on day -1 and day 15. An intraperitoneal glucose tolerance test (IPGTT) was conducted on day 14. On this day, mice received their daily injection of peptide at midday rather than between 16:00 and 17:00. The mice were culled on day 16 via decapitation to allow for collection of large volumes of blood for plasma analysis.

***In vivo* GIPR desensitisation studies**

To measure the effect of a single dose of GIP108 (30 nmol/kg) on GIPR desensitisation, mice were injected at 17:00 prior to an acute food intake study (4-way crossover, n=37-40/group), IPGTT (hGIP (50 nmol/kg), 4-way crossover, n=14-16/group; hGIP (500 nmol/kg), 2-way crossover, n=6-8/group) or insulin bleeds (2-way crossover, n=8/group) conducted the following day.

**Intraperitoneal glucose tolerance tests**

Mice were fasted at 08:00. For all studies, 20% glucose was administered at 14:00, with the injection volume adjusted to the body weight of the mouse such that each mouse received glucose at 2 g/kg. To assess effects of GIP108 on day 14 of the chronic study, mice were dosed with saline or GIP108 at a specified dose at 12:00. To assess the effects of GIP108 on pancreatic GIPR desensitisation, hGIP at a specified dose was administered alongside intraperitoneal glucose injection. Blood glucose measurements were taken via tail vein venesection at baseline, t=15, t=30, t=60, t=90 and t=120 mins. Glucose AUC is calculated using y=0. Glucose readings were measured in mmol/L using the same GlucoRx glucometer.

**Insulin bleeds**

Mice were fasted at 08:00. At 14:00, mice received an intraperitoneal injection with 20% glucose, with the injection volume adjusted to the body weight of the mouse such that each mouse received glucose at 2 g/kg. At this time, some mice were administered hGIP (50 nmol/kg) with glucose as the vehicle. Blood samples were taken via tail vein venesection at baseline and t=15 minutes following injection.

**Acute food intake studies**

To identify a suitable anorectic dose of hGIP, HFD-induced obese mice were fasted at 17:00. Mice were re-fed and injected with vehicle or a specified dose of hGIP at 08:00 the following day. Food intake was measured at t=30, t=60, t=120 and t=240 mins. To assess the effects of GIP108 on CNS GIPR desensitisation the same protocol was followed, with hGIP (200 nmol/kg) being administered at 08:00.

**Echo MRI**

Body composition was measured using an EchoMRI^TM^ machine. This allows for the measurement of lean mass, fat mass and water mass. The mass was recorded in grams. Mice were restrained in a red Perspex tube which was then inserted into the EchoMRI^TM^ machine. Each scan took 160 seconds. The tube was cleaned between animals to minimise stress.

**Plasma assays**

Insulin was quantified following acute hGIP (50 nmol/kg) administration using the Mercodia Mouse Insulin ELISA kit (10-1247-01, Mercodia, Sweden). Leptin, adiponectin and amino acids were quantified following chronic GIP108 administration. Plasma amino acids were measured using the Abcam L-amino acid assay kit (ab65347, Abcam, UK). Plasma adiponectin was measured using the Abcam mouse adiponectin ELISA kit (ab226900, Abcam, UK). Plasma leptin was measured using the Abcam mouse leptin ELISA kit (ab199082, Abcam, UK). All assays were performed according to the manufacturer’s protocol and were read using the SpectraMax i3x Microplate Reader (Molecular Devices, US).

**Islet isolation and dispersion**

Primary cultures of pancreatic islets were prepared from 6–24-week-old male and female mice as described previously [1*]. For dispersion, islets were washed in PBS and dispersed into single cells by trituration in 0.05% trypsin-EDTA for 3 min at 37°C.

**Hypothalamic neuronal isolation**

Primary cultures of hypothalamic neurons were prepared from 6-week-old male and female mice as described previously [2*]. A minor difference was that the neuronal pellet was resuspended in Neurobasal-A medium containing 0.25% Glutamax, 2% B27 and 25 ng/ml NGF (all Thermo Fisher, UK) (neuronal culture media).

**Cell plating and cADDis transduction**

Dispersed islets: A cell suspension of dispersed islets in RPMI-1640 + 10% FBS + 1% P/S (islet complete media) was seeded onto square 96 well µ-plates (Ibidi, UK), coated with 0.01% poly-D-lysine hydrobromide (Sigma-Aldrich, UK) and 25 µg/ml mouse laminin (Thermo Fisher, UK). For cADDis experiments, cells were transduced with the Green Up cADDis biosensor (15 μL of cADDis, 1.5 μL of sodium butyrate per well: Montana Molecular, US). Cells were incubated overnight (37°C, 95%:5% O_2_:CO_2_ ratio).

Hypothalamic neurons: 100 μL cell suspension of neurons in neuronal culture media was seeded on 35 mm petri dish (MatTek Corporation), pre-coated with 0.01% poly-D-lysine hydrobromide. Cells were transduced with cADDis (20 μL of cADDis, 0.4 μL of sodium butyrate per dish). After a 1 hr incubation period (37°C, 95%:5% O_2_ CO_2_ ratio), an additional 2 ml of culture media was added.

**4hr and 24hr GPCR desensitisation experiments**

To study the effects of GIP108, hGIP, liraglutide and GLP-1 on GPCR desensitisation *ex vivo*, pancreatic islets were treated for either 4 hours or 24 hours with specified peptides at specified doses. During this treatment, peptides were dissolved in islet culture media. Prior to imaging, the peptide washout period was 1 hour in Krebs buffer with 6 mM glucose and 0.1% BSA.

**cAMP imaging using cADDis**

Cells were imaged in Krebs buffer with 6 mM glucose and 0.1% BSA (unless 10% FBS was specified) at 37°C using an automated epifluorescence microscope (modified Nikon Ti-2E with LED illumination). Multiple fields of view (FOV) were imaged in parallel (for islets, multiple wells of a 96 well plate were imaged in parallel), allowing hundreds to thousands of cells to be imaged every minute throughout the time course. For all acquisitions, the first three minutes were baseline recording pre-peptide addition and the last three minutes were a positive control following IBMX (500 μM) and forskolin (50 μM) addition to maximally stimulate the sensor.

**cAMP imaging using CAMPER mouse islets**

Cells were imaged in a similar setup described in the previous paragraph. However, an image spitter (OptoSplit III, Cairn Research Ltd, UK) was used to enable simultaneous acquisition of cyan fluorescent protein (CFP) and yellow fluorescent protein (YFP) emission. cAMP was quantified based on the YFP/CFP FRET ratio from each cell.

**cAMP Image analysis**

cAMP quantification from the raw islet/neuronal images was performed using Fiji v1.54f (NIH). For each acquisition, a flat-field (BaSiC [3*]) correction was applied, and intensity thresholding enabled cell-containing regions to be identified as regions-of-interest (ROIs). The fluorescence intensity of each ROI was measured at each time point. In pre-treatment experiments, islet cell fluorescence was normalised to the maximal IBMX/FSK response which was set as 100%. For non-pre-treatment experiments, neuronal and islet cell fluorescence were normalised to both the baseline and maximal IBMX/FSK response. The AUC during peptide stimulation for each cell was quantified. Neurons with an AUC greater than 75 (representing a response 15 % of the maximum IBMX/FSK response) were deemed responder neurons.

**Method references**

[1*] Hinds C, Peace E, Chen S, Davies, I, El Eid L, Tomas A, et al. Abolishing β-arrestin recruitment is necessary for the full metabolic benefits of G protein-biased glucagon-like peptide-1 receptor agonists. Diabetes, Obesity and Metabolism 2024;26(1):65-77

[2*] Adriaenssens AE, Biggs EK, Darwish T, Tadross, J, Sukthanker T, Girish M, et al. Glucose-Dependent Insulinotropic Polypeptide Receptor-Expressing Cells in the Hypothalamus Regulate Food Intake. Cell Metab. 2019;30(5):987-996.e6.

[3*] Peng, T, Schroeder T, Wang L, Theis FJ, Marr C, Navab N*.* A BaSiC tool for background and shading correction of optical microscopy images. Nature communications 2017;8

**Supplementary Note:**

*Dose selection for GIPR agonists*

We aimed to determine appropriate pre-treatment doses of GIPR agonists which would produce detectable cAMP responses, indicating target engagement, and were compatible with an extended incubation in complete medium, which could influence ligand availability via binding to serum proteins. We first performed concentration-response analysis in dispersed islets transduced with cADDis in the presence of 10% FBS **(Figure S2A, B, D, E)**. GIP108 responses were detectable at doses above 100 nM, with a calculated EC_50_ of 1957 nM, compared to 69.43 nM for native GIP. As preincubation effects might be influenced by ligand degradation in extended culture, we tested the stability of hGIP and GIP108 at 1 μM in complete medium in the presence of dispersed islet cells (**Figure S2G, H**). Using production of cAMP in mouse GIPR-expressing AD-293 cells as a bioactivity assay, we determined that GIP108 remained partially stable at 24 hours, with 215 nM remaining from 500 nM (concentrations halved during assay). hGIP remained stable at 4 hours, but not 24 hours, with only 0.14 nM remaining from 500 nM. Based on these findings, 100 nM and 1 μM were chosen as suitable 4-hour pre-treatment doses of hGIP, and doses up to 1 μM were chosen as 4-hour and 24-hour pre-treatment doses of GIP108.

To determine a sensible re-challenge dose of GIPR agonists, a further concentration-response analysis was conducted in cADDis-transduced dispersed islets in imaging buffer (Krebs buffer + 6 mM glucose + 0.1% BSA, i.e. without FBS) (**Figure S2C, F**). This indicated that GIP108 responses were detectable at doses above 10 nM, with a calculated EC_50_ of 217.6 nM, compared to 6.367 nM for native GIP. Therefore, 1 μM of GIP108 and 100 nM of GIP were chosen as peri-maximal re-stimulation doses for the remaining islet cAMP assays.

*Dose selection for GLP-1R agonists*

GLP-1R agonist pre-treatment dose selection was performed concurrently with GIPR agonist dose selection. Concentration-response analysis in dispersed islets transduced with cADDis in the presence of 10% FBS showed that islet cells responded to doses of GLP-1 and liraglutide above 0.1 nM with an EC_50_ of 0.295 nM and 1.041 nM, respectively (**Figure S2A, B, D, E)**. Bioactivity assessment determined that GLP-1 (10 nM) remained stable after 4 hours but not 24 hours incubation with dispersed islet cells in complete medium, with only 0.177 nM remaining from 500 nM. Liraglutide (10 nM) partially degraded after 24 hours incubation with dispersed islet cells in complete medium, with 0.97 nM of liraglutide remaining from 5 nM (concentrations halved during assay) and 0.12 nM of liraglutide remaining from 0.5 nM (**Figure S2G, I**). Therefore, 1 and 10 nM of GLP-1 and liraglutide were considered sensible pre-treatment doses for comparison to 100 nM - 1 μM of GIP and 1 μM of GIP108, respectively.

To determine a sensible re-challenge dose of GLP-1R agonists, we referred to in house concentration response analysis using dispersed islets, conducted in imaging buffer. Islets responded to doses of liraglutide and GLP-1 above 0.1 nM. 1 nM and 10 nM of liraglutide and 1 nM of GLP-1 were chosen as peri-maximal re-stimulation doses for the remaining islet cAMP assays.


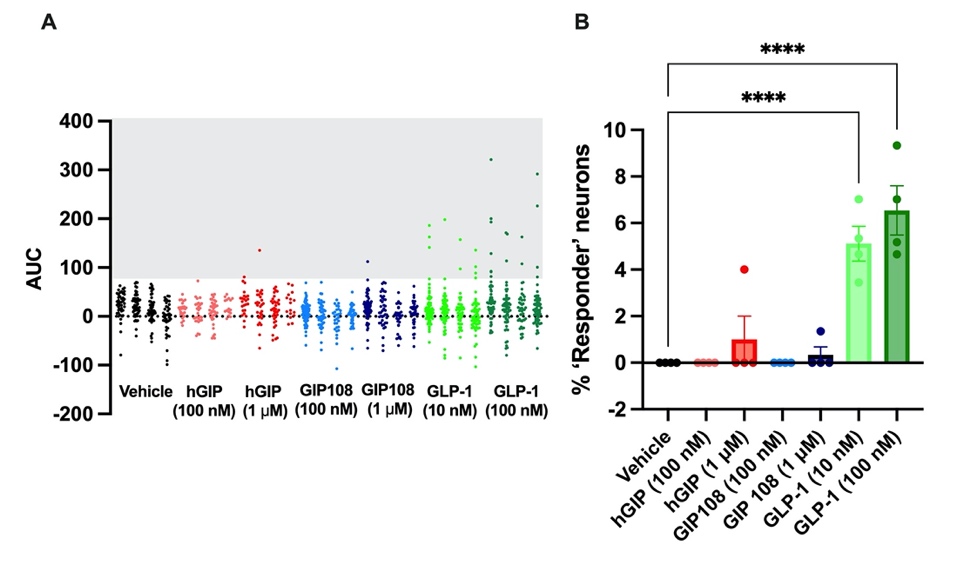


**Supplementary Figure 1: Primary murine hypothalamic neurons do not produce cAMP in response to GIPR agonism**

**A)** The individual AUCs of each neuron during 5-minute stimulation with specified peptides. **B)** The % of hypothalamic neurons whose cAMP profile had an AUC that was >15% of their FSK/IBMX response (contained within grey shading in **A**) and thus deemed a ‘responder’ neuron when stimulated for 5 minutes with the specified peptides (n=4). Data was analysed using a one-way ANOVA. Dunnett’s test was used to correct for multiple comparisons. In **B)** data is presented as mean ± SEM. **** = P<0.0001.


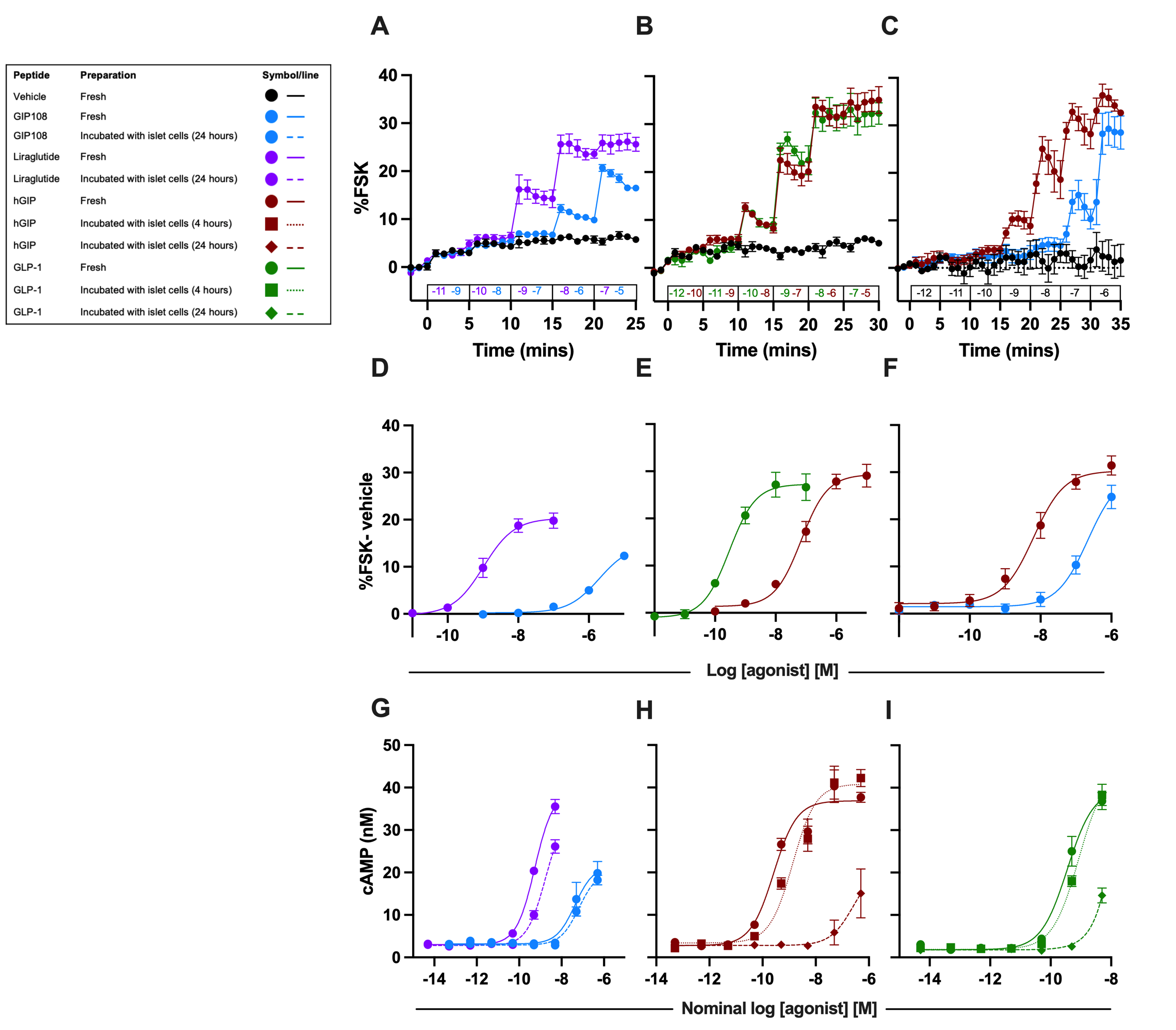


**Supplementary Figure 2: Selection of *in vitro* doses for desensitisation studies**

**A-C)** cAMP signal as a percentage of FSK/IBMX response in dispersed islet cells (transduced with cADDis) in response to stepwise addition of specified peptides (log concentration above the y-axis) (n=5) in 10% FBS (**A-B**) or 0.1% BSA (**C**). **D-F)** cAMP dose response curves derived from the AUC in each time interval in **A-C** minus vehicle control, with three parameter fits shown. **G-I**) cAMP dose responses in AD-293 cells transiently transfected with mouse GLP-1R (for liraglutide and GLP-1 stimulation) and mouse GIPR (for GIP108 and hGIP stimulation). Cells were stimulated for 30 minutes with fresh peptides (n=2) or peptides diluted from agonists (10 nM for liraglutide and GLP-1, 1 μM for hGIP and GIP108) exposed to islet cells for 4 hours or 24 hours (as indicated) (n=4). The nominal agonist concentration represents the calculated stimulatory concentration assuming no degradation. Three-parameter curve fitting was applied. All values are displayed as mean ± SEM.


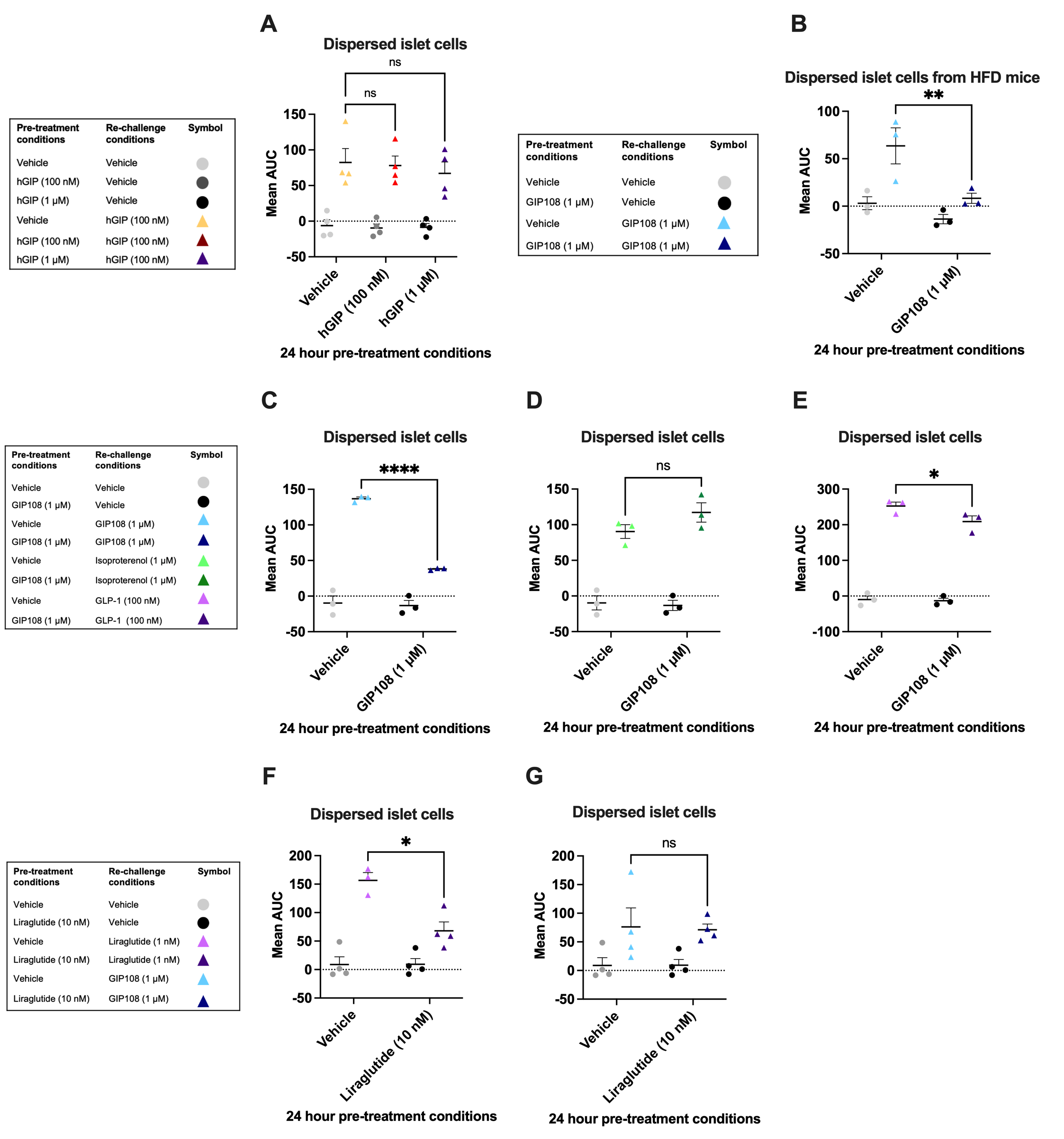


**Supplementary Figure 3: *Ex vivo* islet GIPR desensitisation (continuation from Figure 2)**

**A**) GIPR desensitisation in dispersed pancreatic islet cells (transduced with cADDis) (n=4) following 24-hour pre-treatment with specified doses of hGIP and re-challenged with vehicle or hGIP (100 nM). **B**) GIPR desensitisation in dispersed pancreatic islet cells from HFD-induced obese mice (transduced with cADDis) (n=3) following 24-hour pre-treatment and re-challenge with GIP108 (1 µM). **C-E**) GPCR desensitisation in dispersed pancreatic islet cells (transduced with cADDis) (n=3) following 24-hour pre-treatment with GIP108 (1 µM) and re-challenged with vehicle, GIP108 (1 µM) (**C**), isoproterenol (1 µM) (**D**) and GLP-1 (100 nM) (**E**). **F, G**) GPCR desensitisation in dispersed pancreatic islet cells (transduced with cADDis) (n=3-4) following 24-hour pre-treatment with liraglutide (10 nM) and re-challenged with vehicle, liraglutide (1 nM) (**F**) and GIP108 (1 µM) (**G**). cAMP profiles for all experiments are displayed in **Supplementary Figure 4**. **A-G)** The mean AUC calculated from the cAMP profile for each repeat. Here, the data has been analysed using a two-way ANOVA with pre-treatment groups and re-challenge groups as co-variables. The Šídák test was used to correct for multiple comparisons. All values are displayed as mean ± SEM. * = P<0.05, ** = P<0.01, **** = P< 0.0001.


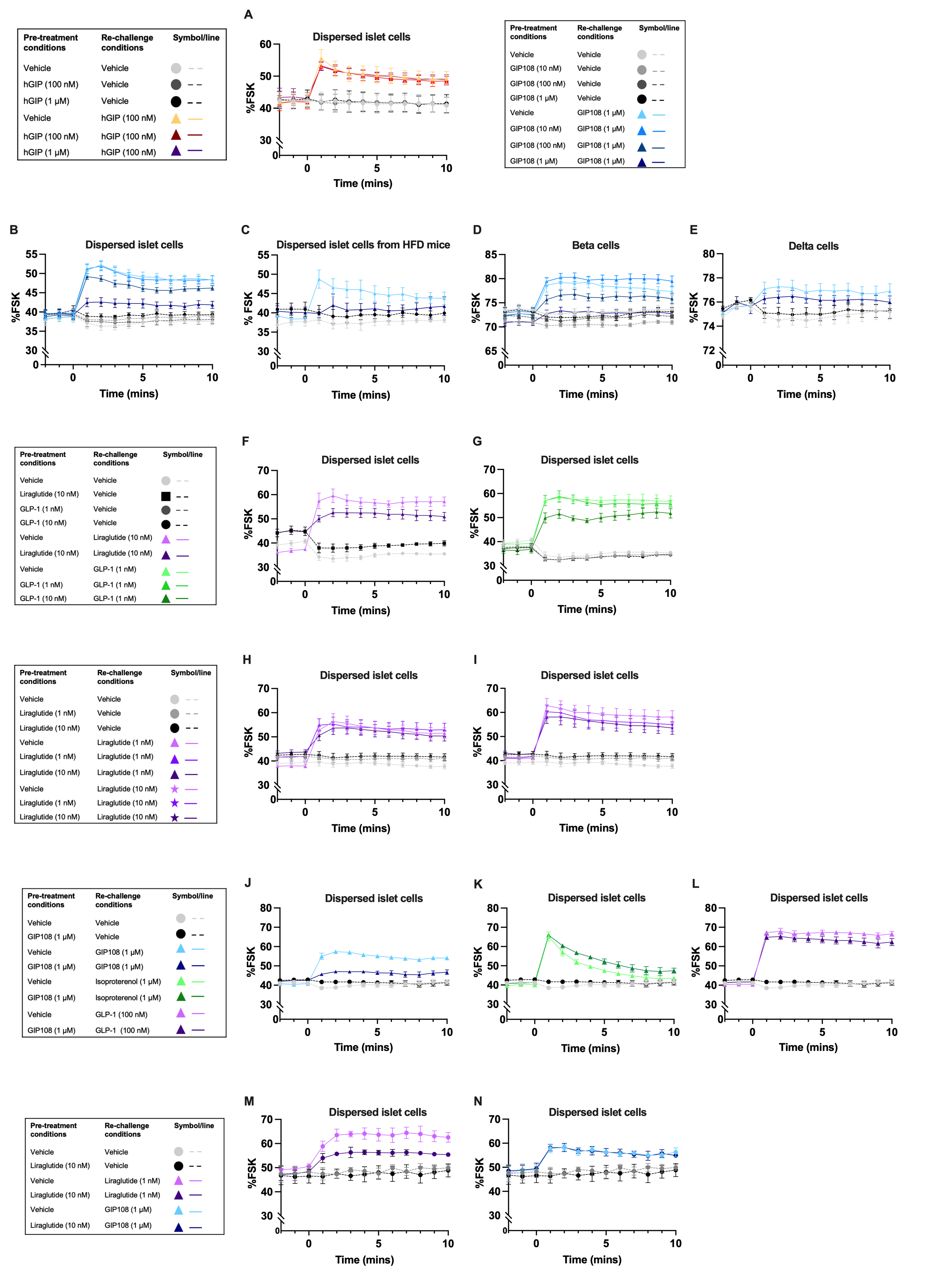


**Supplementary Figure 4: cAMP profiles from *ex vivo* islet GIPR and GLP-1R desensitisation studies**

The cAMP profiles for experiments displayed in **Figure 2** and **Supplementary Figure 3**. They are displayed as a percentage of the maximum cAMP reached with FSK/IBMX treatment following 10 minutes of treatment with either vehicle or specified peptide, with or without pre-treatment with specified peptide. **A)** GIPR desensitisation in all dispersed pancreatic islet cells (transduced with cADDis) (n=4) following 24-hour pre-treatment with specified doses of hGIP. **B-E)** GIPR desensitisation in dispersed pancreatic islets (transduced with cADDis) from lean mice (n=11-12) (**B**), dispersed pancreatic islets (transduced with cADDis) from HFD-induced obese mice (n=3) (**C**), pancreatic beta cells (CAMPER*^Pdx~~-~~1-CreERT2^* ) (n=4) (**D**) and pancreatic delta cells (CAMPER*^Som-Cre^*) (n=5) (**E**) following 24hr pre-treatment with specified doses of GIP108. **F, G)** GLP-1R desensitisation in all dispersed pancreatic islet cells (transduced with cADDis) (n=3) following 4hr pre-treatment with specified doses of liraglutide and GLP-1 and re-challenged with liraglutide (10 nM) (**F**) and GLP-1 (1 nM) (**G**). **H, I)** GLP-1R desensitisation in all dispersed pancreatic islet cells (transduced with cADDis) (n=4-5) following 24hr pre-treatment with specified doses of liraglutide and re-challenged with liraglutide (1 nM) (**H**) and liraglutide (10 nM) (**I**). **J-L**) GPCR desensitisation in dispersed pancreatic islet cells (transduced with cADDis) (n=3) following 24-hour pre-treatment with GIP108 (1 µM) and re-challenged with vehicle, GIP108 (1 µM) (**J**), isoproterenol (1 µM) (**K**) and GLP-1 (100 nM) (**L**). **M, N**) GPCR desensitisation in dispersed pancreatic islet cells (transduced with cADDis) (n=3-4) following 24-hour pre-treatment with liraglutide (10 nM) and re-challenged with vehicle, liraglutide (1 nM) (**M**) and GIP108 (1 µM) (**N**). All values are displayed as mean ± SEM.


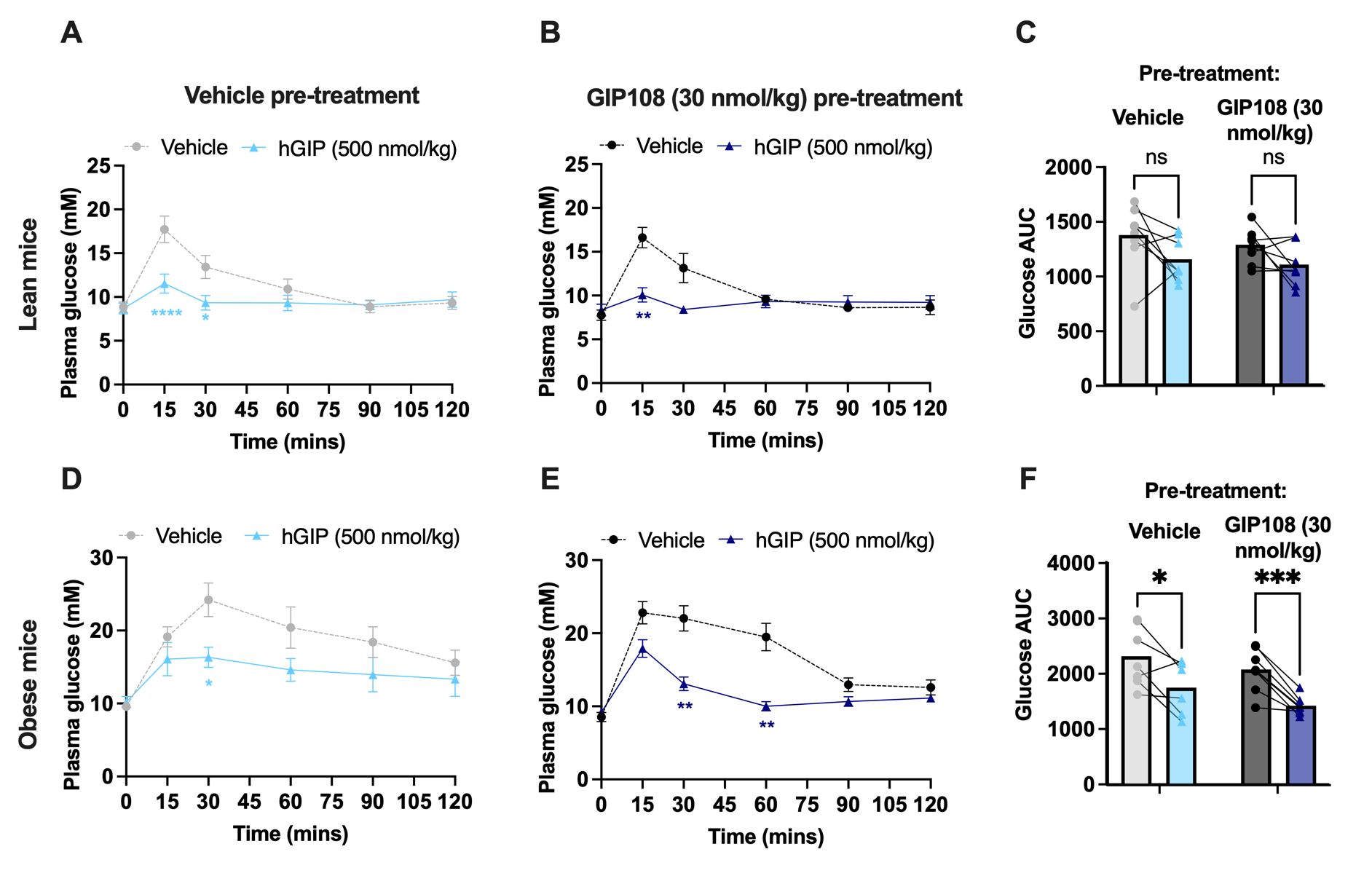


**Supplementary Figure 5: *In vivo* pancreatic GIPR desensitisation is rescued using high concentrations of hGIP**

2-way crossover 120-minute intraperitoneal glucose tolerance tests testing responses to vehicle and hGIP (500 nmol/kg) in lean (**A-C**) (n=8) and HFD-induced obese mice (**D-F**) (n=6-8). **A**, **B**, **D** and **E**) Plasma glucose time course following glucose and peptide injection at t=0 mins. **C** and **F**) Mean glucose AUC generated from the time course. Mice had received a subcutaneous vehicle injection (**A** and **D**) or GIP108 (30 nmol/kg) injection (**B** and **E**) 21 hours prior to the IPGTT. Blood glucose at specific time points have been analysed using a two-way ANOVA with time and subgroup as co-variables. Glucose AUCs have been analysed using a two-way ANOVA with IPGTT treatment and pre-treatment groups as co-variables. The Šídák test was used to correct for multiple comparisons. All values are displayed as mean ± SEM. * = P<0.05, ** = P<0.01, *** = P<0.001, **** = P<0.0001.


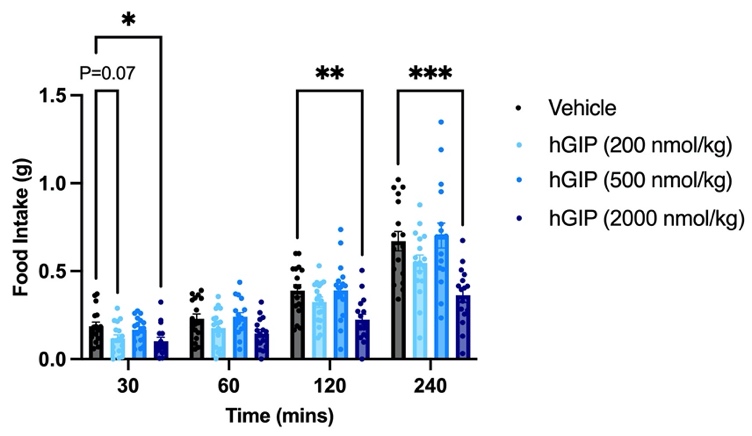


**Supplementary Figure 6: hGIP results in acute reductions in food intake in HFD-induced obese mice**

Acute fast-refeed food intake study in HFD-induced obese mice (n=16-17) testing responses to specified doses of hGIP. Food intake at specific time points have been analysed using a two-way ANOVA with time and subgroup as co-variables. The Dunnett’s test was used to correct for multiple comparisons. All values are displayed as mean ± SEM. * = P<0.05, ** = P<0.01, *** = P<0.001.
